# Numerical and Experimental Analysis of Material Removal and Surface Defect Mechanism in Scratch Tests of High Volume Fraction SiCp/Al Composites

**DOI:** 10.3390/ma13030796

**Published:** 2020-02-10

**Authors:** Xu Zhao, Yadong Gong, Ming Cai, Bing Han

**Affiliations:** 1 School of Mechanical Engineering and Automation, Northeastern University, Shenyang 110819, China; 2 School of Mechanical Engineering and Automation, University of Science and Technology Liaoning, Anshan 114051, China

**Keywords:** SiCp/Al composites, scratch test, 3D finite element model, material removal, surface defect

## Abstract

This paper addresses a comprehensive and further insight into the sensitivity of material removal and the surface defect formation mechanism to scratch depth during single-grit scratch tests of 50 vol% SiCp/Al composites. The three-dimensional (3D) finite element model with more realistic 3D micro-structure, particle-matrix interfacial behaviors, particle-particle contact behaviors, particle-matrix contact behaviors and a Johnson-Holmquist-Beissel (JHB) model of SiC was developed. The scratch simulation conducted at scratch velocity 10 mm/min and loading rate 40 N/min revealed that the scratch depth plays a crucial role in material removal and the surface forming process. Brittle fracturing of SiC particles and surface defects become more deteriorative under a large scratch depth ranging from 0.0385 to 0.0764 μm. The above phenomenon can be attributed to the influence of scratch depth on SiC particles’ transport; the increase in the amount of SiC particle transport resulting from an increase of scratch depth raises the occurrence of particle-particle collision which provides hard support and shock for the scratched particles; therefore, brittle fracturing gradually becomes the major removal mode of SiC particles as the scratch depth increases. On the deteriorative surface, various defects are observed; i.e., lateral cracks, interfacial debonding, cavies filled with residually broken particles, etc. The von Mises stress distribution shows that SiC particles bear vast majority of load, and thus present greater stress than the surrounding Al matrix. For example: their ratio of 3 to 30 under the scratch depth of 0.011 mm. Namely, SiC particles impede stress diffusion within the Al matrix. Finally, the SEM images of the scratched surface obtained from the single-grit scratch experiments verify the numerical analysis’s results.

## 1. Introduction

Particle reinforced metal matrix composites (PRMMCs) own excellent ratios of strength-to-weight; relatively low thermal expansion coefficients; and corrosion and wear resistance. Therefore, they have drawn worldwide attention in transportation, aerospace and defense fields. But as difficult-to-machine materials resulting from non-homogeneity properties between hard SiC and soft metal matrix, PRMMCs present various surface and subsurface defects after machining operations, especially high volume fraction PRMMCs [1]. In order to achieve good surface and subsurface quality, grinding has been introduced as a relatively effective processing method for those PRMMCs, but extensive grinding-induced damages still exist in high volume fraction SiCp/Al. Systematic improvement of grinding quality, such as by parameter optimization, needs a comprehensive understanding of the material removal mechanism of high volume fraction SiCp/Al composites [2].

Single-grit scratch tests are commonly used to explain the material removal process during grinding process. A few researchers conducted single-grit scratch experiments to study material removal of SiCp/Al composites. Feng et al. [3], Zha et al. [4] and Zheng et al. [5] conducted ultrasonic vibration-assisted and traditional scratch experiments on SiCp/Al composites which indicated that ultrasonic vibration could benefit SiC removal and improve the scratched surface. These references well indicate that single-grit scratch tests can represent grinding to reveal material removal of PRMMCs. However, conventional scratch experiments only present consequential phenomena, such as surface morphology and scratch force, which could not intuitively track a dynamic micro-material removal process, which includes tool-reinforcements interaction, tool-matrix interaction, reinforcement-matrix interfacial debonding evolution, crack propagation and material deformation; thus, it is impossible to reveal the underlying material removal mechanism of PRMMCs via conditional experiments. Fortunately, such a limitation is able to be overcome by numerical simulations.

Recently, a number of PRMMC machining models have been developed. Zhu et al. [6] established a two-dimensional (2D) orthogonal cutting model of 10 vol% alumina/6061 aluminum composites, in which reinforcement-matrix interface failure was modeled via multiple point constraints, the weak reinforcement-matrix interfacial effect was negligible and reinforcements’ removal was not incorporated. The results indicated that different reinforcement-tool conditions caused fluctuations of chip-tool interfacial stress. Pramanik et al. [7] studied the effect of reinforcement-tool interaction positions on tool wear, matrix deformation and particle debonding via a 2D orthogonal cutting model, where the position and shape of reinforcements were regular, and weak reinforcement-matrix interfacial effect was also negligible. Wang et al. [8] established 2D orthogonal cutting models of SiCp/Al composites to study surface defect formation mechanism of 65 vol% SiCp/Al composites, in which weak reinforcement-matrix interfacial effect was also negligible. They pointed that SiC fracture states played a major role in the machined surface defect. Zhou et al. [9] studied the surface and edge topography, and the subsurface residual stress of different cutting parameters via a 2D orthogonal cutting model of 55 vol% SiCp/Al, where weak reinforcement-matrix interfacial effect was also negligible. Teng et al. [10] investigated the effect of size scale of reinforcements (nano-particles and micro-particles) on chip formation and surface topography via 2D orthogonal cutting models of 10 vol% SiCp/Al composites; in their model, the position and shape of reinforcements were regular, and weak reinforcement-matrix interfacial effect was also negligible. They found that nano-particles were beneficial for improvement of the machined surface. Wang et al. [11] established a 3D orthogonal cutting model of SiCp/Al to study the effect of removal process of SiC reinforcements on surface generation; the results showed that the positional relation of reinforcements and tools played a leading role in SiC fracture behaviors which resulted in different surface defects. Tn their model, the position and shape of SiC were regular and unreal, and the weak reinforcement-matrix interfacial effect was also negligible. Umber et al. [12] compared the cutting forces of PRMMCs via orthogonal cutting models with and without cohesive zone elements utilized for the particle-matrix interface. The results showed that interfacial cohesive behavior modeling was essential for PRMMCs machining simulations. However, the random position and shape of reinforcements and its fracture behavior were lacking. Ghandehariun et al. [13,14,15] utilized 2D orthogonal cutting models of 10 vol% Al_2_O_3_p/Al with reinforcement-matrix cohesive behaviors to conduct a series of studies, including tool-workpiece interactions and the effect of cutting speed on such interactions; in their models, the position and shape of reinforcements were regular.

Based on the above literature, the summarization of recent simulation studies of PRMMC machining is shown in Table 1. In these studies, composite structure models of PRMMCs were all simplified, which did not fully take into account the randomness of reinforced particle sizes, shapes and positions in 3D space, or the weak particle-matrix interfacial effects; thus, the validity of these machining simulations is problematic. Those machining simulations were all based on orthogonal cutting models with cutting tools of positive rake, which are suitable for turning and milling, but not suitable for scratching and grinding with grits of negative rake. Therefore, in spite of the advances achieved in PRMMC machining simulations, the more reasonable modelling of the PRMMC scratch process still remains blank and challenging.

The study aims to track dynamic material removal and defect the surface formation process of high volume fraction SiCp/Al composites during single-grit scratch tests via a more realistic 3D finite element (FE) model, in which the study realizes the randomness of reinforced particle sizes, shapes and positions in 3D space; additionally, weak particle-matrix interfacial effects, particle-particle contact and particle-matrix contact behaviors are taken into account. The simulation results are finally validated by single-grit scratch experiments. The simulation and experiment of single-grit scratch provides theoretical guidance and the key parameters optimal for the grinding of SiCp/Al in real applications.

Additionally, the simulation technology of scratch tests in the study will also promote behavioral studies of coatings applied for cutting tools, which are widely being conducted by more and more researchers, such as Rodriguez-Barrero [16], Fernandez-Abia [17] and Fernandez-Valdivielso [18].

## 2. Materials and Methods

### 2.1. Specimen

**Figure 1:**
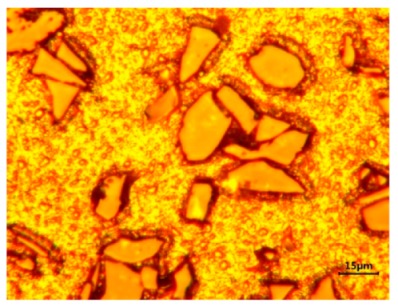
The microstructure of 50 vol% SiCp/5083Al.

In order to satisfy the urgent request of the opto-mechanical structure for grinding quality of SiCp/Al composites (40–50 vol%), material removal and the surface defect mechanism were analyzed via single-grit scratch tests of 50 vol% SiCp/Al composites, which were prepared by powder metallurgy technique, in which the average diameter of reinforced particle was 20 μm. Firstly, mixing of Al5083 powders and SiC particles were sufficiently conducted by ball milling at 150 rpm for 10 h with the weight ratio of 27:32, and the mixture was put into a mold for cold isostatic compaction. It was then heated in a vacuum furnace HIP-200 at 753 K for 2 h under 120 Mpa, and followed by cooling with the furnace. Finally, the extradition of the composite was conducted at 773 K with an extrusion ratio of 20:1; then, the composite was held at 833 K for 3 h and artificially aged at 423 K for 18 h. Figure 1 presents the microstructures of the composites. The dimensions of the polished specimen are 40 mm in length, 35 mm in width and 15 mm in height.

### 2.2. Finite Element Modelling

**Figure 2:**
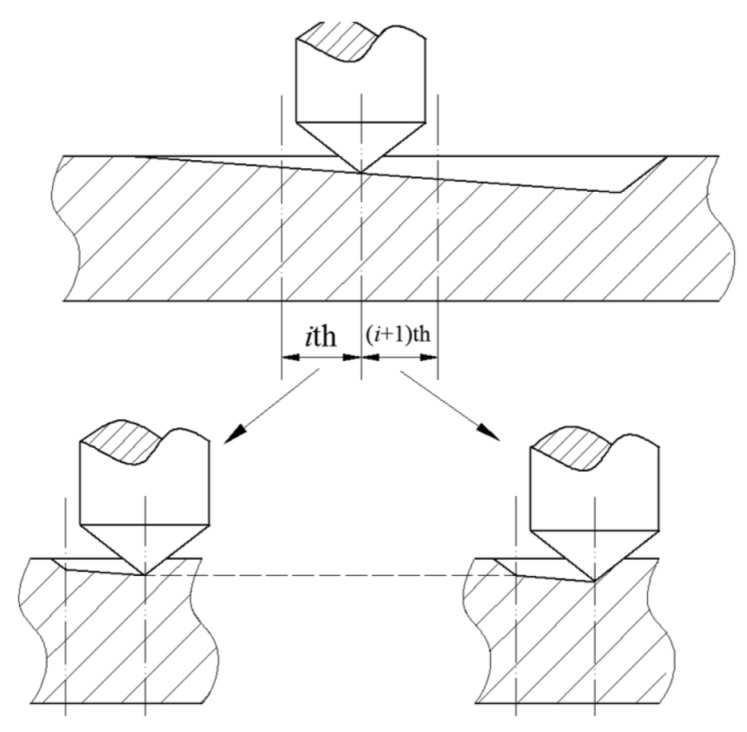
Schematic diagram of the scratch process simulation.

3D FE scratch models of 50 vol% SiCp/Al composites were established via Abaqus/Explicit 2017; the scratch process is as follows: The indenter with cone angle 120° and tip radius 20 μm exerts an increasing load on the specimen. In the meantime, the specimen moves with uniform motion, and then a gradually deepening scratched groove appears on the specimen’s surface. The process is divided into 3 sequential sub-processes to reduce computation time, as shown in Figure 2. The selected scratch parameters are listed in Table 2.

#### 2.2.1. Microscopic Structural Modeling and Mesh of SiCp/Al Composites

**Figure 3:**
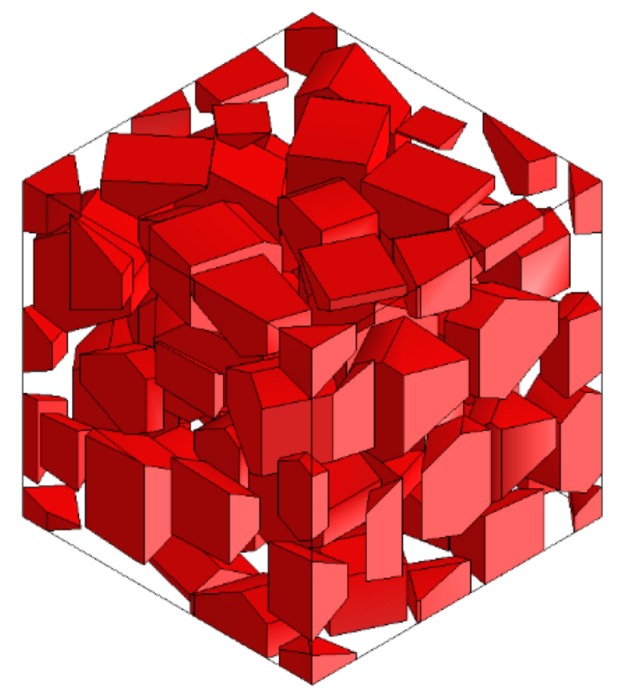
3D structural model of SiC/Al of 100 × 100 × 100 μm^3^.

SiC particles own polyhedral structures, and particles shapes, sizes and positions in the Al matrix are all random. Based the above structural characteristics of reinforced particles, a program package was developed to achieve geometric modeling of various polyhedral particles with random sizes and shapes, and random distributions of particles positions within a prescribed scope; namely, the volume fraction of SiC particles is about 50%, and particles sizes satisfy a normal distribution of mean 20 μm and variance 5 μm. An established 3D geometry model of SiCp/Al composite (100 × 100 × 100 μm^3^) for the first two sub-processes is shown in Figure 3, and the size of the SiCp/Al composite for the third sub-process is 200 × 200 × 200 μm^3^.

**Figure 4:**
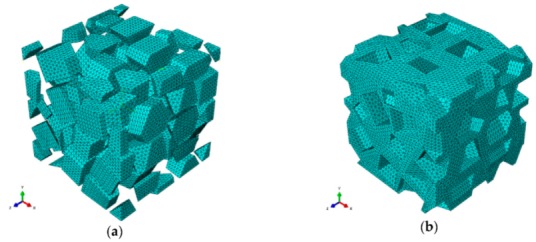
The meshing strategy for SiC particles and Al matrix: (**a**) SiC particles, (**b**) Al matrix.

Free 10-node modified thermally coupled second-order tetrahedron meshing technique and seed size of 3 μm were adopted to generate mesh for SiC particles and Al matrix, as shown in Figure 4.

#### 2.2.2. Material Properties of Al Matrix, SiC Particle and Diamond Intender

Table 3 presents the basic mechanical properties of SiC particles, 5083Al matrix and the diamond indenter. The Johnson Cook constitutive model and Johnson Cook damage law, which are universally used in metal machining FE simulations, were adopted to describe 5083Al matrix. The Johnson Cook constitutive model is described as follows [19]:

(1) $$\bar{\sigma }=\left[A+B{\left({\bar{\varepsilon }}^{pl}\right)}^{n}\right]\left[1+C\ln\left(\frac{{\dot{\bar{\varepsilon }}}^{pl}}{{\dot{\varepsilon }}_{0}}\right)\right]\left[1-{\left(\frac{T-T_{\mathrm{room}}}{T_{\mathrm{melt}}-T_{\mathrm{room}}}\right)}^{m}\right]$$

where $\bar{\sigma }$, ${\bar{\varepsilon }}^{pl}$, ${\dot{\bar{\varepsilon }}}^{pl}$ and ${\dot{\varepsilon }}_{0}$ denote equivalent flow stress, plastic strain, plastic strain rate and reference plastic strain rate respectively; T, $T_{\mathrm{room}}$ and $T_{\mathrm{melt}}$ are the material real-time, room and melting temperature respectively; additionally, five material constants are as follows: A—initial yield strength, B—strain hardening modulus, C—strain rate sensitivity coefficient, n—strain hardening coefficient and m—thermal softening coefficient. The Johnson Cook constitutive model parameters for 5083Al are listed in Table 4.

The Johnson Cook damage law is given by

(2) $${\bar{\varepsilon }}_{f}^{pl}=\left(D_{1}+D_{2}e^{D_{3}\eta }\right)\left[1+D_{4}\ln\left(\frac{{\dot{\bar{\varepsilon }}}^{pl}}{{\dot{\varepsilon }}_{0}}\right)\right]\left[1+D_{5}\left(\frac{T-T_{\mathrm{room}}}{T_{\mathrm{melt}}-T_{\mathrm{room}}}\right)\right]$$

where $D_{1}$–$D_{5}$ are material constants; ${\dot{\varepsilon }}_{0}$ and η are reference strain rate and pressure to von Mises ratio. Those parameters for 5083Al are presented in Table 5.

When the damage parameter ω=1, which is calculated in Equation (3), fracture occurs.

(3) $$\omega =\sum \frac{\Delta {\bar{\varepsilon }}^{pl}}{{\bar{\varepsilon }}_{f}^{pl}}$$

where $\Delta {\bar{\varepsilon }}^{pl}$ is the equivalent plastic strain increment.

In all studies of SiCp/Al machining so far, a linearly elastic constitutive model with damage initiation has been adopted for SiC particles. However, this study describes SiC particles by the Johnson-Holmquist-Beissel (JHB) model which is more suitable for SiC responses under large strain and high-strain rate [20]. The strength of the undamaged SiC (damage variable, D=0) is given by Equation (4).

(4) $$\{\begin{array}{c} \sigma =\left(1+C\ln{\dot{\varepsilon }}^{*}\right)\left[\sigma_{i}\left(P+T\right)/\left(P_{i}+T\right)\right]\;\;\mathrm{if}\;P\leq P_{i}\;\\ \sigma =\left(1+C\ln{\dot{\varepsilon }}^{*}\right)\left\{\sigma_{i}+\left(\sigma_{i}^{\max}-\sigma_{i}\right)\left\{1-\exp\left[-\alpha_{i}\left(P-P_{i}\right)\right]\right\}\;\right\}\;\mathrm{if}\;P\geq P_{i}\; \end{array}$$

where $\alpha_{i}=\sigma_{i}/\left[\left(\sigma_{i}^{\max}-\sigma_{i}\right)\left(P_{i}+T\right)\right]$ and $P_{i}$, T, $\sigma_{i}$, $\sigma_{i}^{\max}$ and C are material parameters; ${\dot{\varepsilon }}^{*}$ is the dimensionless equivalent strain rate, ${\dot{\varepsilon }}^{*}={\dot{\varepsilon }}^{pl}/{\dot{\varepsilon }}_{0}$ (where ${\dot{\varepsilon }}^{pl}$ and ${\dot{\varepsilon }}_{0}$ are the equivalent plastic strain rate and ${\dot{\varepsilon }}_{0}$ is the reference strain rate, respectively); and P is a pressure function expressed in Equation (6). The strength of the fractured SiC (D=1) is given by Equation (5).

(5) $$\{\begin{array}{c} \sigma =\left(1+C\ln{\dot{\varepsilon }}^{*}\right)\left[\sigma_{f}P/P_{f}\;\right]\;\;\;\mathrm{if}\;P\leq P_{f}\;\\ \sigma =\left(1+C\ln{\dot{\varepsilon }}^{*}\right)\left\{\sigma_{f}+\left(\sigma_{f}^{\max}-\sigma_{f}\right)\left\{1-\exp\left[-\alpha_{f}\left(P-P_{f}\right)\right]\right\}\right\}\;\mathrm{if}\;P\geq P_{f} \end{array}$$

where $\alpha_{f}=\sigma_{f}/\left[\left(\sigma_{f}^{\max}-\sigma_{f}\right)P_{f}\right]$ and $P_{f}$, $\sigma_{f}$ and $\sigma_{f}^{\max}$ are material parameters.

The pressure function P is described as Equation (6).

(6) $$\left\{ \begin{array}{l} P=K_{1}\mu +K_{2}\mu^{2}+K_{3}\mu^{3}-K_{1}\mu_{f}+\sqrt{{\left(K_{1}\mu_{f}\right)}^{2}+2\beta K_{1}\Delta U}\;\mathrm{if}\;\mu \geq 0\;and\;\mu_{f}>0\\ P=K_{1}\mu +K_{2}\mu^{2}+K_{3}\mu^{3}\;\;\;\mathrm{if}\;\mu \geq 0\;and\;\mu_{f}\leq 0\\ P=K_{1}\mu \;\;\;\;\;\mathrm{if}\;\mu \leq 0 \end{array}\right.$$

where $\mu =\rho /\rho_{0}-1$, $K_{1}$, $K_{2}$ and $K_{3}$ are constants; $\rho_{0}$ and ρ denote the reference and current density respectively; $\mu_{f}$ equals μ at the time of failure; β is the ratio of the elastic energy loss; and ΔU denotes the dilation increment.

The damage parameter, ω, is expressed as Equation (7).

(7) $$\omega =\sum \frac{\Delta {\bar{\varepsilon }}^{pl}}{{\bar{\varepsilon }}_{f}^{pl}\left(P\right)}$$

where $\Delta {\bar{\varepsilon }}^{pl}$ denotes the equivalent plastic strain increment and ${\bar{\varepsilon }}_{f}^{pl}\left(P\right)$ is given by

(8) $${\bar{\varepsilon }}_{f}^{pl}=D_{1}{\left(P^{*}+T^{*}\right)}^{D_{2}}\;{\bar{\varepsilon }}_{f,\min}^{pl}\leq {\bar{\varepsilon }}_{f}^{pl}\leq {\bar{\varepsilon }}_{f,\max}^{pl}$$

where $D_{1}$ and $D_{2}$ are material constants and $P^{*}=P/\sigma_{i}^{\max}$ and $T^{*}=T/\sigma_{i}^{\max}$. ${\bar{\varepsilon }}_{f,\min}^{pl}$ and ${\bar{\varepsilon }}_{f,\max}^{pl}$ are the minimum and maximum fracture strain respectively.

In the JHB model, SiC particles suffer failure immediately when ω=1; otherwise, (ω<1) there is no damage. The JHB model material parameters used for SiC are listed in Table 6.

The diamond indenter was treated as an analytical rigid body because of its high elastic modulus. The material parameters applied in the FE computational analysis are listed in Table 3.

#### 2.2.3. Particle-Matrix Interfacial Modeling

The interfacial behaviors between SiC particles and an Al matrix play a crucial role in material removal and the surface defect formation processes. Because of tiny dimensions, the interfacial thickness is ignored. Two kinds of interfacial behaviors are considered in the study: (i) a cohesive interface which may be damaged and fail; (ii) a friction interface which occurs after failure of the cohesive interface. The cohesive interfacial behaviors are achieved via cohesive behavior model and damage model. The friction interfacial behaviors are realized via tangential behavior model and normal behavior model in the general contact type of Abaqus/Explicit 2017.

The cohesive behavior model specifies that only salve nodes initially in contact experience cohesive behaviors, and it uses the traction-separation model which is defined by Equation (9).

(9) $$t=\left\{\begin{array}{c} t_{n}\\ t_{s}\\ t_{t} \end{array}\right\}=\left[\begin{array}{ccc} K_{n} & 0 & 0\\ 0 & K_{s} & 0\\ 0 & 0 & K_{t} \end{array}\right]\left\{\begin{array}{c} \delta_{n}\\ \delta_{s}\\ \delta_{t} \end{array}\right\}=K\delta$$

where t denotes the nominal traction stress vector consisting of normal $t_{n}$, $\mathrm{shear}\;t_{s}$ and $\mathrm{shear}\;t_{t}$; the corresponding separations are $\delta_{n}$, $\delta_{s}$ and $\delta_{t}$. According to Lotfian, S. [21], the undamaged stiffnesses $K_{n}$, $K_{s}$ and $K_{t}$ are all set as 1 × 10^12^ MPa⋅mm^−1^.

The cohesive damage model includes damage initiation specified by the cohesive interfacial strength $t^{0}$ and damage evolution specified by the fracture energy Γ. As for particle-matrix cohesive interfacial strength $t^{0}$, Guo [24] designed a novel experiment to measure interfacial strength $t^{0}=133\pm 26\;\mathrm{MPa}$; and Nan [25] and Su [26] reasonably figured out that the cohesive interfacial strength followed approximately $t^{0}\sim 1/d^{1/2}$, where d is the average particle size; thus, while the average size is 20 μm, $t^{0}=138$ MPa, and fracture energy is set as Γ=91.9 J/m^2^. Obviously, the two above conclusions are very close; therefore, the cohesive interfacial strength is set as $t^{0}=t_{n}^{0}=t_{s}^{0}=t_{t}^{0}=133\;\mathrm{MPa}$, and Γ=0.0919 mJ/mm^2^ in the work.

On friction interfacial behaviors, Coulomb’s friction law with friction coefficient μ=0.3 is used to model sliding conditions for tangential behavior model, and hard contact is selected as pressure-overclosure for normal behavior model.

#### 2.2.4. Particle-Particle, Indenter-Particle and Indenter-Matrix Contact Modeling

Regular contact interfaces were adopted for particle-particle, indenter-particle and indenter-matrix interactions; Coulomb’s friction law was used to model sliding conditions for tangential behavior and hard contact for normal behavior; the friction coefficient μ=0.4 was used for particle-particle contact; μ=0.1 was used for indenter-particle contact; and μ=0.15 was used for indenter-matrix contact.

#### 2.2.5. Loads and Boundary Conditions

**Figure 5:**
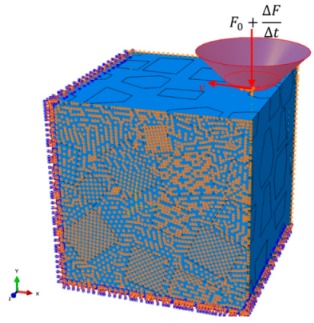
Loads and boundary conditions.

In Figure 5, the indenter constrained with the reference point RF could only move along x-direction at v=−10 mm/min and along y-direction at a normal force $F=-\left(F_{0}+\frac{\Delta F}{\Delta t}\right)$, where $F_{0}$ and $\frac{\Delta F}{\Delta t}$ are the initial normal force and increment per unit time, respectively.

As shown in Figure 5, the bottom and front faces of the SiCp/Al 3D model are constrained in 6-Degree of Freedom(DOF), $U_{x}=U_{y}=U_{z}={\mathrm{UR}}_{x}={\mathrm{UR}}_{y}={\mathrm{UR}}_{z}=0$, while the z-direction of its two lateral faces is fixed,${\;V}_{Z}=0$.

### 2.3. Single-Grit Scratch Experiments

**Figure 6:**
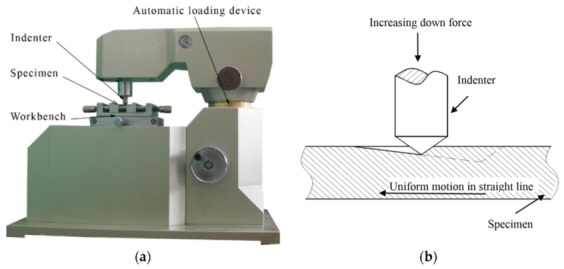
Experimental method: (**a**) MFT-4000 Scratch Tester for Material Surface Properties; (**b**) schematic diagram of scratch with a linearly increasing down force.

The experiments were conducted on MFT-4000 Scratch Tester for Material Surface Properties, which as developed by Lanzhou Huahui Instrument Technology Co., Ltd. (Lanzhou, China), via using a conical diamond indenter with cone angle 120° and tip radius 20 μm (see Figure 6a). Table 7 shows the main technical indicators of MFT-4000 Scratch Tester. An automatic loading device exerts an increasing load on the specimen via the indenter; in the meantime, the specimen moves with a uniform motion, and then a gradually deepening scratched groove appears on the specimen’s surface, as illustrated in Figure 6b. The selected scratch parameters are listed in Table 2. The scratched surface morphology was observed via ZEISS Ultra Plus Field Emission Scanning Electron Microscope (SEM, ZEISS, Jena, Thuringia, Germany).

## 3. Results and Discussion

### 3.1. Material Removal Process

Multiple factors affect the material removal process in scratch tests of high volume fraction SiCp/Al composite, such as indenter-particle contact, particle-matrix interfacial behavior and particle motion within the matrix, which are all directly influenced by the scratch depth.

#### 3.1.1. The Initial Scratch Process

**Figure 7:**
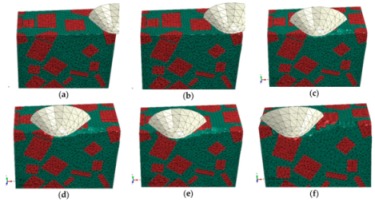
Simulated initial scratch process at a depth of 0 to 0.011 mm: (**a**) scratch depth *h* = 0, (**b**) *h* = 1.01 × 10^−5^ mm, (**c**) *h* = 3.96 × 10^−3^ mm, (**d**) *h* = 5.77 × 10^−3^ mm, (**e**) *h* = 8.43 × 10^−3^ mm, (**f**) *h* = 0.011 mm.

**Figure 8:**
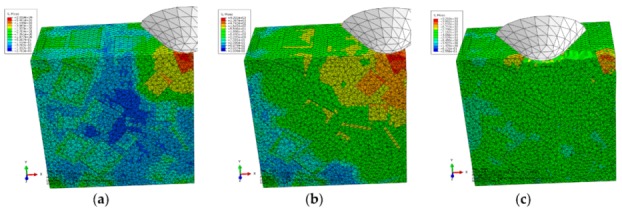

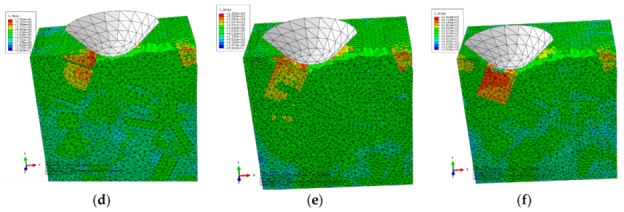
Von Mises stress distribution of the initial scratch model sectioned along the scratch direction: (**a**) scratch depth *h* = 0, (**b**) *h* = 1.01 × 10^−5^ mm, (**c**) *h* = 3.96 × 10^−3^ mm, (**d**) *h* = 5.77 × 10^−3^ mm, (**e**) *h* = 8.43 × 10^−3^ mm, (**f**) *h* = 0.011 mm.

**Figure 9:**
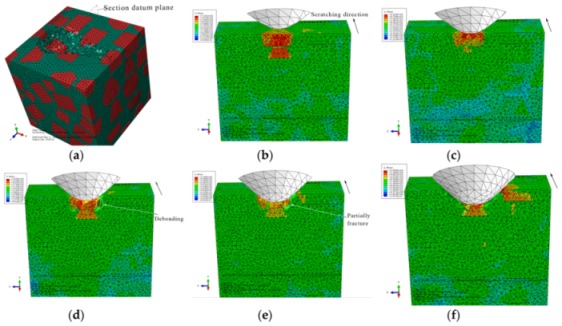
Von Mises stress distribution on the initial scratch model sectioned perpendicularly to the scratch direction: (**a**) Sectioning scheme, (**b**) *t* = 0.294 s, (**c**) *t* = 0.315 s, (**d**) *t* = 0.336 s, (**e**) *t* = 0.356 s, (**f**) *t* = 0.419 s.

During the initial scratch process under the normal load of 0 to 5 N and the scratch depth of 0 to 0.011 mm, the model is sectioned along the scratch direction for material removal analysis, as shown in Figure 7. When the scratch depth is very small and the indenter scratches only the SiC particle, the SiC particle and Al matrix endure elastic deformation; they revert to their original state after the indenter scratches into the Al matrix from the SiC particle. The material removal is negligible (see Figure 7a,b). As the scratch depth increases, the Al matrix is removed in ductile mode when the indenter scratches the Al matrix (see Figure 7c). When the indenter scratches into the SiC particles from the Al matrix, a SiC particle first rotates on a small scale and moves toward the lower left because of the indenter’s negative rake, and then heads toward other SiC particles. As the indenter advances, the Al matrix between the approaching SiC particles endures squeezing and damage failure, and the SiC particle removal occurs mainly in the ductile mode (see Figure 7d,e), but partly in the brittle fracture mode when a particle crashes into an another particle (see Figure 8d,e). After the particle is completely removed, the indenter scratches some other particles (see Figure 7f and Figure 9f); the indenter is in contact with some particles almost all the time due to high volume fraction of particles in the Al matrix.

The von Mises stress distributions in the longitudinal section along the scratch direction and the cross section perpendicular to the scratch direction during the initial scratch models are shown in Figure 8 and Figure 9, respectively. Because of high hardness, SiC particles bear the vast majority of load; thus, they present greater stress than the surrounding Al matrix. As shown in Figure 8, in the longitudinal section, von Mises stress diffuses from the workpiece region pressed by the indenter tip to the lower left because of the indenter’s negative rake. The particles in the lower left impede stress diffusion; therefore, SiC particles equivalently enhance the anti-deformation ability of the Al matrix.

Figure 8 reveals four situations: (1) The indenter scratches a SiC particle; the particle experiences highly concentrated stress (see Figure 8a,e,f). (2) The indenter scratches only the Al matrix; the stress in the scratched area of the Al matrix is of a larger amplitude, and accumulated stress is spread to the particles in the lower left (see Figure 8c). (3) The indenter scratches into the Al matrix from the SiC particle; the stress of the Al matrix increases with a large amplitude as that of the SiC particle decreases (see Figure 8b). (4) The indenter scratches into the SiC particle from the Al matrix; a high localized stress zone is found at the indenter-particle contact area when the indenter first engages with the particle (see Figure 8d). As the indenter advances, the maximum localized stress zone with larger amplitude moves to the left side of the particles, which leads to the damage initiation, and the stress is transferred to an another particle in the lower left via the Al matrix, and then the particle scratched by the indenter is removed gradually and mainly in the ductile mode. The Al matrix between the two approaching particles is continuously squeezed to deform seriously and finally be removed with advancement of the indenter (see Figure 8e).

On the cross section shown in Figure 9, the stress is transferred from the workpiece region pressed by the indenter tip to the bottom, left and right sides, respectively. As indenter advances, the stress on the particle contacting the indenter tip increases gradually; the particle is pushed down toward to an another particle (see Figure 9b,c); then, the Al matrix between the two approaching particles is squeezed to be removed, and the particle mainly experiences ductile removal (see Figure 9c,d) and slight brittle fracturing (see Figure 9e). A very small portion of particle-matrix interfacial debonding (see Figure 9d) occurs because of the particle rotation phenomenon, which is also observable in Figure 7d,e. During the process, the indenter pushes the particles aside; these particles are removed in ductile mode because of the small scratch depth (see Figure 9e,f).

**Figure 10:**
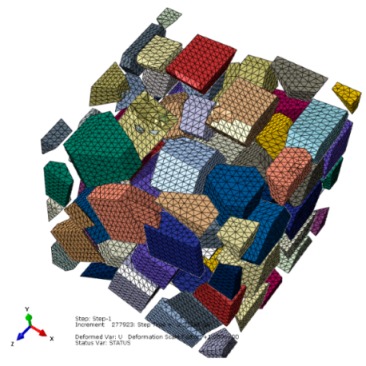
The final state of SiC particles in the initial scratch model.

During the initial scratch process under the scratch depth of 0 to 0.011 mm, SiC particles and Al matrix are primarily removed in ductile mode; brittle fracturing of SiC particles rarely occurs because of small scratch depth and the flexible support provided by Al matrix which is beneficial to ductile removal of SiC particles. Moreover, a large amount of particle-matrix interfacial failure and particle-particle collision do not appear due to minor migration of SiC particles. The final state of SiC particles is shown in Figure 10.

#### 3.1.2. The Middle Scratch Process

**Figure 11:**
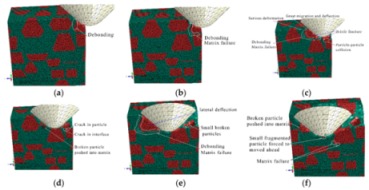
Simulated middle scratch process at a depth of 0.011 to 0.0385 mm: (**a**) *h* = 0.011 mm, (**b**) *h* = 0.0122 mm, (**c**) *h* = 0.0162 mm, (**d**) *h* = 0.0212 mm, (**e**) *h* = 0.0290 mm, (**f**) *h* = 0.0385 mm.

During the middle stage of scratch process under the normal load of 5 to 12 N and the scratch depth of 0.011 to 0.0385 mm, the model is sectioned along the scratch direction for material removal analysis, as shown in Figure 11. As the scratch depth increases from 0.011 to 0.0385 mm, not only does SiC-Al interfacial debonding becomes more evident and widespread, but matrix failure around the interface and particle-particle collision does too (see Figure 11a,b,d,e). It is clear that brittle fracturing of particles is more severe when particle-particle collisions occur (see Figure 11c), because a particle-particle collision has a dramatic impact on each particle. With the indenter advances, the particle is pushed to experience great migration and (lateral) deflection, which result in serious deformation and failure of Al matrix and interfacial debonding (see Figure 11c,e). During the process, some broken particles are pushed into Al matrix (see Figure 11d,f) while some small SiC fragments under the indenter tip are possibly pushed ahead on the scratched surface (see Figure 11f). Moreover, microcracks widely exist on particles and the particle-matrix interface (see Figure 11d).

**Figure 12:**
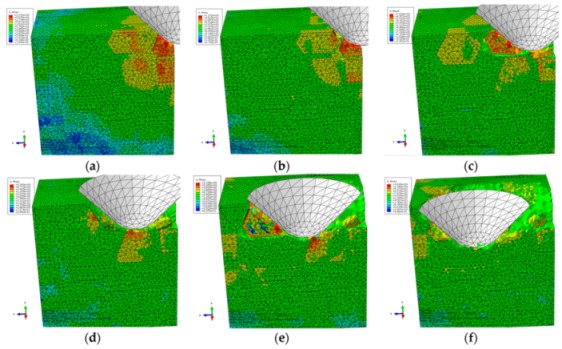
Von Mises stress distribution on the middle scratching model sectioned along the scratching direction: (**a**) *h* = 0.011 mm, (**b**) *h* = 0.0122 mm, (**c**) *h* = 0.0162 mm, (**d**) *h* = 0.0212 mm, (**e**) *h* = 0.0290 mm, (**f**) *h* = 0.0385 mm.

**Figure 13:**
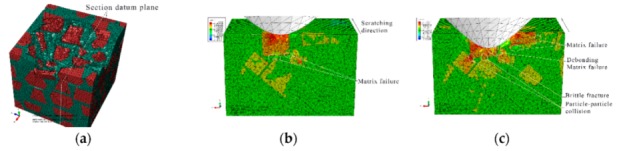

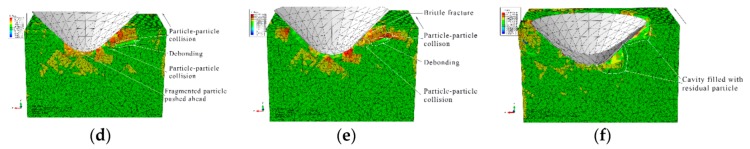
Von Mises stress distribution on the middle scratching model sectioned perpendicularly to the scratching direction: (**a**) sectioning scheme, (**b**) *t* = 0.157 s, (**c**) *t* = 0.209 s, (**d**) *t* = 0.235 s, (**e**) *t* = 0.261 s, (**f**) *t* = 0.365 s.

The von Mises stress distributions in the longitudinal section along the scratch direction and the cross section perpendicular to the scratch direction during the middle scratch process are shown in Figure 12 and Figure 13, respectively. The characteristics of von Mises stress distributions on the two sections are similar to those during the initial scratch process; namely, SiC particles bear the vast majority of load and the greatest stress, and impede stress transmission in the Al matrix. Von Mises stress in the longitudinal section diffuses from the region pressed by the indenter tip to the lower left (see Figure 12), and von Mises stress in the cross section is transferred from the region pressed by the indenter tip to the bottom, left and right sides (see Figure 13). As shown in Figure 12 and Figure 13, the overall von Mises stress level of the scratched zone in various stages of the middle scratch process is relatively higher than that of the initial scratch process, which illustrates the deteriorative material removal process, including the interfacial debonding, brittle fracturing of SiC particles, serious deformation and failure of the Al matrix and particle-particle collision.

Figure 13 also reveals the lateral material removal process: the particles underneath the indenter tip are pushed down to another particle, while the particles on the left or right side of the tip are pushed aside to impact other particles. During the process, failure of Al matrix between the approaching particles and particle-matrix interfacial debonding occurs, followed by particle-particle collision which causes the brittle fracturing of particles (see Figure 13b,c). With the indenter advances, the above phenomena become more evident, and some fragmented particles are pushed ahead by the indenter (see Figure 13d,e). With some fragmented particles pushed ahead, cavities filled with residual particle fragments emerge (see Figure 13f).

**Figure 14:**
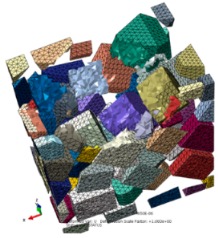
The final state of SiC particles in the middle scratching model.

During the middle scratching process at a depth of 0.011 to 0.0385 mm, ductile removal and brittle fracturing of SiC particles equally occur; meanwhile, some terrible phenomena, i.e., particle-matrix interfacial debonding, serious deformation of Al matrix and particle-particle collision, become more common, and cavities filled with residual particle fragments appear. Because of greater migration of SiC particles, the cluster of particles becomes more evident; the final state of SiC particles is shown in Figure 14.

#### 3.1.3. The Final Scratch Process

**Figure 15:**
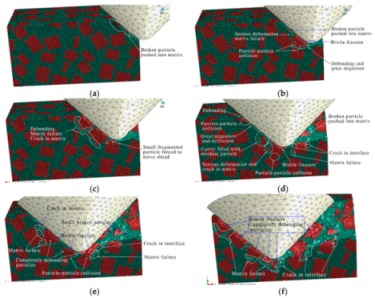
Simulated final scratching process at a depth of 0.0385 to 0.0764 mm: (**a**) *h* = 0.0385 mm, (**b**) *h* = 0.0442 mm, (**c**) *h* = 0.0476 mm, (**d**) *h* = 0.0571 mm, (**e**) *h* = 0.0654 mm, (**f**) *h* = 0.0764 mm.

During the final stage of scratch process under the normal load of 12 to 20 N and the scratch depth of 0.0385 to 0.0764 mm, the model is sectioned along the scratch direction for material removal analysis, as shown in Figure 15. Based on observations in Figure 15, the deteriorative material removal process becomes more evident with the increasing scratching depth, including particle-particle collision, brittle fracturing of particles, interfacial debonding, serious deformation and failure of the Al matrix and cavities filled with residual particles, and so on. Additionally, large-scale cracks in SiC-Al interface become widespread (see Figure 15e,f). The above phenomenon is the result of the larger scratch depth, under which particles are forced to move and rotate on a large scale; then, particle-particle collision and particle-matrix interfacial debonding consequentially occur and become universal during the process (see Figure 15b–e). On account of the high pressure applied by the indenter and the impact effect produced by particle-particle collision, brittle fracturing is the primary mode of SiC material removal (see Figure 15b,d–f). With the indenter advances, the broken particles are pushed into the Al matrix (see Figure 15a,b,d), and particles of complete debonding are forced to move ahead (see Figure 15e,f). Due to severe and irreversible plastic deformation of the Al matrix around the particle-particle collision area, failure and cracking of the Al matrix also frequently appear (see Figure 15c,e,f). Because of the non-uniform deformation between the SiC particles and the Al matrix, when the particle is forced to move or rotate on a large scale, which both result from a large scratch depth, the interfacial debonding evolves into cracks in the interface (see Figure 15f) which further result in large-scale lateral cracks on the scratched surface. That will be discussed in the next section. During the process, cavities filled with residual particles are also observed, as shown in Figure 15d.

**Figure 16:**
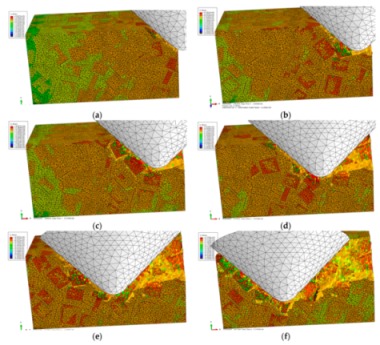
Von Mises stress distribution on the middle scratching model sectioned along the scratching direction: (**a**) scratching depth *h* = 0.0385 mm, (**b**) *h* = 0.0442 mm, (**c**) *h* = 0.0476 mm, (**d**) *h* = 0.0571 mm, (**e**) *h* = 0.0654 mm, (**f**) *h* = 0.0764 mm.

**Figure 17:**
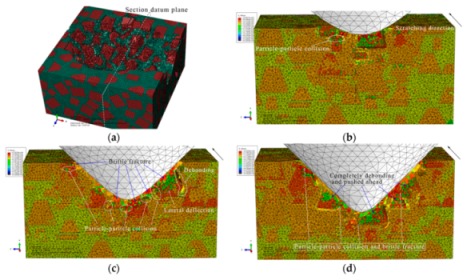

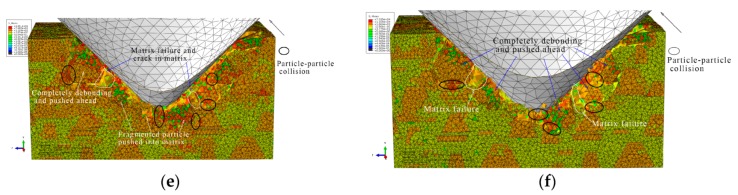
Von Mises stress distribution on the final scratching model sectioned perpendicularly to the scratching direction: (**a**) sectioning scheme, (**b**) *t* = 0.445 s, (**c**) *t* = 0.594 s, (**d**) *t* = 0.693 s, (**e**) *t* = 0.842 s, (**f**) *t* = 0.941 s.

The von Mises stress distributions in the longitudinal section along the scratch direction and the cross section perpendicular to the scratch direction during the final scratch process are shown in Figure 16 and Figure 17, respectively. The characteristics of von Mises stress distributions in the longitudinal section and the cross section during the final scratch process are similar to those during the initial and middle scratch process, but the difference is that the von Mises stress in the Al matrix is higher and closer to the von Mises stress in SiC particles during the final stage because the impeding effect of SiC particles on stress transmission in the Al matrix weakens with the increasing scratch depth.

The lateral material removal process can be investigated in detail via observation in Figure 17 during the final stage of scratch process. The remarkable increase of particle transport distance within the Al matrix that results from the increasing scratch depth leads to widespread occurrence of particle-particle collision (see Figure 17b–f). Then brittle fracturing of SiC particles and interfacial debonding become common phenomena (see Figure 17c,d). With the indenter advances, broken particles of complete debonding are pushed ahead (see Figure 17d–f), along with failure of and cracks in the Al matrix (see Figure 17e,f), and large-scale cracks on particle-matrix interfaces which will be discussed in the next section.

**Figure 18:**
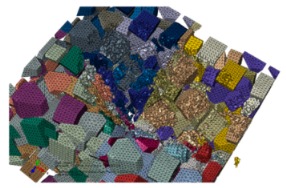
The final state of SiC particles in the final scratching model.

During the final scratch process under the scratch depth of 0.0385 to 0.0764 mm, brittle fracturing is the primary mode of SiC material removal; meanwhile, the deteriorative phenomena, such as particle-particle collision, particle-matrix interfacial debonding, failure and cracks in the Al matrix, long-distance transport of particles and large-scale cracks on the particle-matrix interface, become evident. In particular, large-scale cracks on the particle-matrix interface are the major cause of lateral cracks on the scratched surface, which will be discussed in the next section. Because of the large-scale transport of SiC particles, clusters of particles become more evident during the final scratching process. The final state of SiC particles is shown in Figure 18.

### 3.2. The Scratched Groove Topography

**Figure 19:**
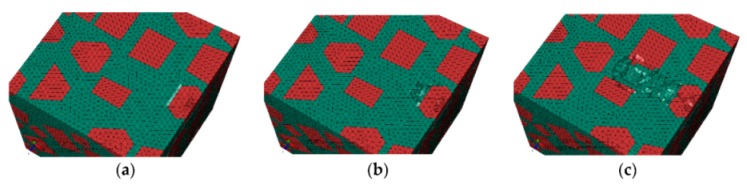

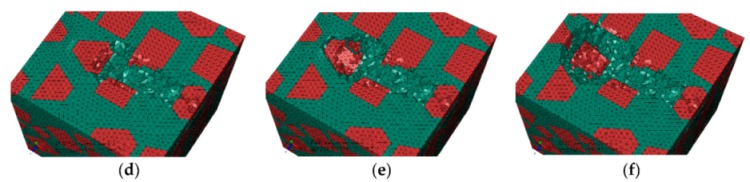
Forming process of the scratched groove topography during the initial scratching stage at a depth of 0 to 0.011 mm: (**a**) scratching depth *h* = 1 × 10^−5^ mm, (**b**) *h* = 3.843 × 10^−5^ mm, (**c**) *h* = 0.00246 mm, (**d**) *h* = 0.00486 mm, (**e**) *h* = 0.00702 mm, (**f**) *h* = 0.011 mm.

The scratched groove topography is a result of the material removal process. Figure 19 shows the formation process of the groove topography during the initial scratch stage under the scratch depth of 0 to 0.011 mm; SiC particles are primarily removed in ductile mode. It is clear that the scratched groove has a good surface quality with very few defects.

**Figure 20:**
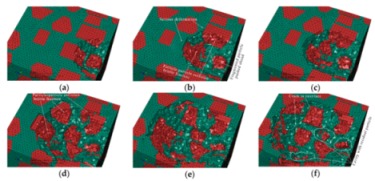
Forming process of the scratched grooves topography during the middle scratching stage at a depth of 0.011 to 0.0385 mm: (**a**) scratching depth *h* = 0.0139 mm, (**b**) *h* = 0.0177 mm, (**c**) *h* = 0.0194 mm, (**d**) *h* = 0.0254 mm, (**e**) *h* = 0.03 mm, (**f**) *h* = 0.0385 mm.

As the scratch depth increases from to 0.011 to 0.0385 mm during the middle scratch stage, particle-particle collision becomes evident, which induces brittle fracturing to become the major removal mode of SiC particles, while the Al matrix between the approaching particles heavily deforms; these phenomena can be seen in Figure 20. During the process, particles are broken into large pieces and small fragments which are pushed into the Al matrix. Because of non-uniform deformation between SiC particles and Al matrix, interfacial debonding appears on the surface, which evolves into cracks on interfaces. In additional, some broken particle pieces are occasionally pushed out by the indenter to form cavities filled with residual particles. Consequently, the scratched surface is considerable coarse, consisting of cracks on the particle-matrix interface and cavities filled with residual particles, as shown in Figure 20f.

**Figure 21:**
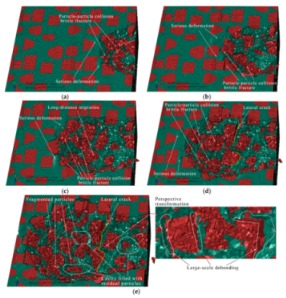
Forming process of the scratched grooves topography during the final scratching stage at a depth of 0.0385 to 0.0764 mm: (**a**) scratch depth *h* = 0.0427 mm, (**b**) *h* = 0.0476 mm, (**c**) *h* = 0.0571 mm, (**d**) *h* = 0.0629 mm, (**e**) *h* = 0.0764 mm.

The final formation process of the scratched groove under the scratch depth of 0.0385 to 0.0764 mm is shown in Figure 21. Particle-particle collision, brittle fracturing of SiC particles and deteriorative deformation of the Al matrix become more serious and common. It is worth noting that cracks on particle-matrix interface evolve into large-scale lateral cracks, which are one of major surface defects (see Figure 21d,e). The evolution process is as follows: The broken particles are pushed ahead by the indenter, and then cracks (debonding) on the particle-matrix interface occur due to non-uniform deformation between SiC particles and matrix. Cracks grow with continuous transport of particles while the surrounding matrix material is torn apart, and lastly, cracks connect to form the lateral crack on the scratched surface. The scratched surface quality becomes more deteriorated with the increase of scratch depth. In addition, the indenter tends to press the particles into the scratched surface, which induces the severe deformation of Al matrix surrounded by particles. Based on the observations of the final scratched surface (see Figure 21e), most of defects on the scratched surface occur in or around the SiC particles, such as lateral cracks, cavities filled with residual particles, particle fragments remaining in the Al matrix and particle-matrix interfacial debonding. So the removal mode of SiC particles plays an important role in the scratched surface formation, and that is allied to scratch depth; namely, the removal mode of SiC particles is almost ductile removal under a small scratch depth, but is brittle fracturing under a large scratch depth.

### 3.3. Experimental Verification

**Figure 22:**
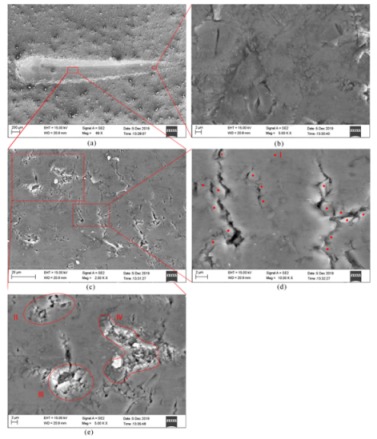
SEM images of the single single-grit scratched groove: (**a**) the overall surface topography, (**b**) the magnified view of the identified area on the initial scratching surface, (**c**) the magnified view of the identified area on the final scratching surface, (**d**) the magnified view of lateral cracks in (**c**) and (**e**) the magnified view of other defects in (**c**). I—the SiC particle which is marked with a red dot; II—small SiC fragments pushed ahead and pressed into the matrix; III—a cavity filled with residual particles; IV—fragmented particles remaining in the matrix.

The scratched surface microstructures of the single-grit scratch experiments performed on the MFT-4000 Scratch Tester were observed by ZEISS Ultra Plus Field Emission Scanning Electron Microscope (SEM), as shown in Figure 22. Figure 22a presents the whole scratched groove; Figure 22b shows that the initial scratched surface under the scratch depth of 0 to 0.011 mm is considerably smooth and exhibits very few defects. The SiC particles are primarily removed in ductile mode. As shown in Figure 22c, the final scratched surface quality under the scratch depth of 0.011 to 0.0385 mm becomes very deteriorative and coarse, on which various surface defects are observed; i.e., lateral cracks, small SiC fragments pushed ahead and then pressed into the Al matrix, cavies filled with residually broken particles, fragmented particles remaining in the matrix and interfacial debonding. It is worth noting that lateral cracks are one of the primary defects. In order to analyze the formation mechanism of lateral cracks, a certain zone of the final scratched surface is magnified in Figure 22d where the SiC particles are marked with red dots. The lateral cracks initiate at several particle-matrix interfacial debonding sites (micro-cracks), and then grow through the matrix as the indenter advances. These interfacial micro-cracks ultimately link together to evolve into large-scale lateral cracks. In the final scratch stage, most of particles are broken into small pieces and/or large pieces which induce various surface defects, after some pieces are removed from the workpiece, leaving lots of cavities filled with residually broken particles, or fragmented particles remaining in the matrix. In addition, removed small SiC fragments are occasionally pushed ahead under the indenter top and then pressed into the matrix. These phenomena can be observed in Figure 22e. Obviously in Figure 22, particle-matrix interfacial debonding on the final scratching surface is almost universal, but one kind of defect, namely, cavities without any residual SiC fragments due to complete pushing out of a SiC particle, barely occurs on the single-grit scratched surface. Nevertheless, that is one of the major defects on turning and milling a SiCp/Al surface [9,12]. The phenomenon is attributed to the difference between the grit with a negative rake and turning (milling) tool with a positive rake.

## 4. Conclusions

Based on the more accurate single-grit scratch model with a more realistic 3D micro-structure, particle-matrix interfacial behaviors, particle-particle contact behaviors, particle-matrix contact behaviors and the Johnson-Holmquist-Beissel (JHB) model of SiC, the material removal process and the surface defect formation mechanism of the 50 vol% SiCp/Al composite at various scratch depths were investigated. SEM images of the scratched groove obtained from a corresponding experiment provide good verification. The numerical and experimental studies allow us to draw the following conclusions:

(1) The scratch depth plays a crucial role in the material removal process. SiC particles are primarily removed in ductile mode under a small scratch depth ranging from 0 to 0.011 mm, and then brittle fracturing of SiC particles becomes more evident with an increase of the scratch depth. It is eventually exhibited as the primary removal model under a large scratch depth ranging from 0.0385 to 0.0764 μm. The above phenomenon is attributed to transport of SiC particles within the Al matrix. Small-scale transport of SiC particles induced by a small scratch depth barely results in particle-particle collision; in this case, SiC particles are mainly sustained by the Al matrix which provides a flexible support that is beneficial to ductile removal of SiC particles. The increase of SiC particle transport with scratch depth raises the occurrence of particle-particle collision, which provides a hard support and shock for the scratched particles; therefore, brittle fracturing gradually becomes the major removal mode of SiC particles as the scratch depth increases. The Al matrix is removed in ductile mode during the whole scratch process.
(2) The removal model of SiC particles plays a significant role in the deformation of the scratched surface. If ductile removal of SiC particles is predominant, the scratched surface is considerably smooth and exhibits very few defects, whereas if brittle fracturing of SiC particles occurs more prevalently, the deteriorative and coarse surface becomes more significant, on which various surface defects are observed; i.e., particle-matrix interfacial debonding, lateral cracks, small SiC fragments pushed ahead and then pressed into the matrix, cavies filled with residually broken particles and fragmented particles remaining in the matrix.
(3) Numerical and experimental analyses both reveal that lateral cracks are one of primary surface defects, which were barely referred in previous simulation literature. The formation mechanism of the lateral cracks is as follows: the lateral cracks initiate at several particle-matrix interfacial debonding sites (micro-cracks), and then grow through the matrix as the indenter advances; these interfacial micro-cracks ultimately link together to evolve into large-scale lateral cracks. The formation process simulation of lateral cracks was performed successfully in the study.
(4) A defect, cavities without any residual SiC fragments due to complete pushing out of a SiC particle, barely occurs on the single-grit scratched surface, while that is one of major defects on turning and milling SiCp/Al surface [9,12], which is attributed to the difference between the grit with a negative rake and turning (milling) tool with a positive rake. This indicates that grinding is more beneficial to improving the processed surface quality of PRMMCs than turning and milling.
(5) The von Mises stress distribution shows that SiC particles bear the vast majority of load; thus, they present greater stress than the surrounding Al matrix. Namely, they impede stress diffusion within the Al matrix.
(6) The SEM images of the scratched surface obtained from the single-grit scratch experiments verify the numerical analysis. Due to the importance of scratch depth for SiC particles removal and surface quality, it can be suggested that a relatively small scratch depth be applied to improve surface quality.

## Figures and Tables

**Table 1:** Recently simulation studies of Particle reinforced metal matrix composite (PRMMC) machining.

Study	Machining Mode Type	Modeling of Reinforced Particles	Particle-Matrix Interfacial Modeling	Partilce-Particle Contact
Zhu [6]	2D micro-orthogonal cutting	Geometric model: polygon, random size and position Material model: - Volume fraction: low	Perfectly bonded with tied node, no interfacial debonding	No
Pramanik [7]	2D micro-orthogonal cutting	Geometric model: circle, constant size and regular position Material model: perfectly elastic material without fracture Volume fraction: 20%	Perfectly bonded with tied node, debonding by Al matrix element failure	No
Wang [8]	2D micro-orthogonal cutting	Geometric model: circle/polygon, random size and position Material model: perfectly elastic material with normal stress fracture criterion Volume fraction: 65%	Perfectly bonded with tied node, debonding by Al matrix element failure	No
Zhou [9]	2D micro-orthogonal cutting	Geometric model: polygon, random size and position Material model: perfectly elastic material with brittle cracking model Volume fraction: 55%	Perfectly bonded with tied node, no interfacial debonding	No
Teng [10]	2D micro-orthogonal cutting	Geometric model: circle, constant size and regular position Material model: perfectly elastic material with brittle cracking model Volume fraction: 10%	Perfectly bonded with tied node, no interfacial debonding	No
Wang [11]	3D micro-orthogonal cutting	Geometric model: sphere, constant size and regular position Material model: perfectly elastic material with brittle cracking model Volume fraction: 20%	Perfectly bonded with tied node, debonding by Al matrix element failure	No
Umer [12]	2D micro-orthogonal cutting	Geometric model: circle, constant size and regular position Material model: linear elastic without considering fracture Volume fraction: 20%	Cohesive zone elements, debonding by cohesive zone elements failure	No
Ghandehariun [13,14,15]	2D micro-orthogonal cutting	Geometric model: circle, constant size and regular position Material model: perfectly elastic material with brittle cracking model Volume fraction: 10%/20%	Perfectly bonded with tied node, debonding by Al matrix element failure	No

**Table 2:** Scratch parameters.

Scratch length	5 mm
Scratch velocity	10 mm/min
Loading rate	40 N/min
maximum load	20 N

**Table 3:** Basic mechanical properties of reinforced SiC particles, 5083Al matrix and diamond.

	SiC [20]	5083Al [21]	Diamond [22]
Young’s modulus (MPa)	420,000	70,000	650,000
Poisson’s ratio	0.14	0.3	0.25
Density (tonne/mm^3^)	3.13 × 10^−9^	2.71 × 10^−9^	1.19 × 10^−8^
Thermal conductivity (mJ/s/mm/K)	81	173	35
Thermal expansion (K−1)	4.90 × 10^−6^	2.36 × 10^−5^	4.00 × 10^−6^
Thermal specific heat (Mj/T/K)	4.27 × 10^8^	9.1 × 10^8^	15 × 10^8^
Inelastic heat fraction	0.9	0.9	0.9

**Table 4:** Johnson Cook constitutive model parameters for 5083Al [23].

*A* (MPa)	*B* (MPa)	*n*	*m*	$T_{\mathrm{melt}}\;\left(K\right)$	$T_{\mathrm{room}}\;\left(K\right)$	C	${\dot{\varepsilon }}_{0}$
167	596	0.551	0.859	893	293	0.001	1

**Table 5:** Johnson Cook damage law parameters for 5083Al [23].

$D_{1}$	$D_{2}$	$D_{3}$	$D_{4}$	$D_{5}$	${\bar{u}}_{f}^{pl}$
0.0261	0.263	−0.349	0.147	16.8	2.1×10^−5^

**Table 6:** JHB model material parameters used for SiC [20].

**Line 1**	$\rho_{0}$ **(tonne/mm^3^)**	G **(MPa)**	$\sigma_{i}$ **(MPa)**	$P_{i}$ **(MPa)**	$\sigma_{f}$ **(MPa)**	$P_{f}$ **(MPa)**	C	${\dot{\varepsilon }}_{0}$
	3.13 × 10^−9^	1.93 × 10^5^	4.92 × 10^3^	1.5 × 10^3^	1 × 10^2^	2.5 × 10^2^	0.009	1.0
**Line 2**	T **(MPa)**	$\sigma_{i}^{\max}$ **(MPa)**	$\sigma_{f}^{\max}$ **(MPa)**	β	$D_{1}$	$D_{2}$	${\bar{\varepsilon }}_{f,\min}^{pl}$	**FS**
	7.5 × 10^2^	1.22 × 10^2^	2 × 10^2^	1.0	0.16	1.0	999	0.2
**Line 3**	$K_{1}$ **(MPa)**	$K_{2}$ **(MPa)**	$K_{3}$ **(MPa)**	**-**	**-**	**-**	**-**	**-**
	2.2 × 10^5^	3.61 × 10^5^	0	-	-	-	-	-

**Table 7:** The main technical indicators of MFT-4000 Scratch Tester for Material Surface Properties.

Loading Mode	Automatically Load
Loading range	0.25 N~200 N automatic loading continuously, the precision is 0.25 N
Scratch length	2 mm~40 mm
Scratch velocity	10 mm/min
Loading rate	1 N/min~100 N/min
Measuring range	0.5 μm~30 μm
Friction measuring range	10 N~100 N, precision is 0.25 N
